# Examining the Feasibility of Smart Blood Pressure Home Monitoring: Advancing Remote Prenatal Care in Rural Appalachia

**DOI:** 10.1089/tmr.2020.0021

**Published:** 2021-03-24

**Authors:** Jennifer D. Runkle, Maggie M. Sugg, Sena McCrory, Carol C. Coulson

**Affiliations:** ^1^ North Carolina Institute for Climate Studies, North Carolina State University, Asheville, North Carolina, USA.; ^2^ Department of Geography and Planning, Appalachian State University, Boone, North Carolina, USA.; ^3^ Department of Obstetrics and Gynecology, Mountain Area Health Education Center, Asheville, North Carolina, USA.

**Keywords:** pregnancy, blood pressure, remote monitoring, mobile health, rural

## Abstract

**Background:**

Hypertensive disorders of pregnancy are a leading cause of U.S. maternal morbidity and mortality. Home blood pressure (BP) monitoring can provide early detection of hypertension (HTN) outside of routine prenatal visits. Yet little is understood about how well self-monitored BP performs during pregnancy, particularly in rural America.

**Objective:**

To examine the feasibility and patient adherence to a self-monitoring BP program and to remotely collect data on pregnant women during the third trimester at a rural health clinic.

**Materials and Methods:**

A repeated-measures prospective design was used to remotely monitor home BP readings. We examined retention and persistence of weekly BP monitoring in late-stage pregnancy, differences between weekly self-monitored and clinic BP measures, the performance of self-monitored BP in early detection of pregnancy-induced HTN, and receptivity to technology-enabled prenatal monitoring.

**Results:**

A total of 30 women enrolled. Women reported high satisfaction with prenatal care, but missed 5 out of 13 clinic visits (54%). Women contributed an average of 31.2 days of home BP monitoring. Findings showed that home systolic and diastolic BP readings slightly varied from clinic readings. Women reported high health-related internet use and e-health literacy. Participants (93%, *n* = 25) reported a willingness to change their behavior during pregnancy in response to personalized recommendations from a smartphone. Although preliminary, we confirmed that remote monitoring can detect elevated BP earlier than in routine clinic visits.

**Conclusion:**

Findings from this study can be used to inform a novel remote monitoring protocol to improve pregnancy care in a rural care setting.

## Introduction

*Hypertensive disorders of pregnancy (HDP)*, including chronic hypertension (HTN), gestational HTN, and preeclampsia, are the leading causes of maternal and perinatal morbidity complications and mortality affecting ∼6–8% of gestations in the United States.^1^ A recent national study in the United States showed that delivery hospitalizations involving preeclampsia and eclampsia were highest among youngest and oldest women, African Americans, in high-poverty areas, and predominate in the south (42% vs. 38% other regions).^2^ With early diagnosis and treatment, HDP-related maternal morbidity and mortality are largely preventable. The primary diagnostic screening marker for HDPs is blood pressure (BP) measurement during pregnancy, which requires repeat physician visits and, in more extreme cases, emergency department or inpatient monitoring. Rapid technical advancements in wearable m-health devices now extend the capabilities of clinical health care monitoring and have significant potential to improve early detection of pregnancy hypertensive complications.

Wearable sensors, smart textiles, and other mobile health (i.e., m-health) innovations present exciting new opportunities to enhance the diagnosis, clinical monitoring, and management of pregnancy health outside traditional care settings.^3–6^ In the area of women's health, smart technology has already been used to motivate weight loss, improve patient compliance in self-managing chronic disease care, including diabetes, heart disease, breast cancer, and osteoporosis, and to support mental health.^7,8^ m-Health technologies may be particularly beneficial to aid in the reduction of pregnancy health disparities and barriers to care in rural and medically underserved areas.^1^ Yet widespread integration of these technologies into routine obstetrics and gynecology practice has remained limited, particularly in a rural health care setting.

Medical care is shrinking in rural areas across the nation. The situation for obstetric care is even direr, with recent estimates showing that less than half of rural hospitals provide obstetric care.^9,10^ The diminishing obstetric care landscape aggravated by existing disparities in access to and availability of health services in rural America presents remote patient monitoring as an especially appealing new patient-centered model to expand access to prenatal care. Based on our preliminary work, technology-enabled prenatal care has surfaced as a viable solution for pregnant women in rural Appalachia, especially women at risk or already diagnosed with hypertensive-related disorders during pregnancy.^6^ Women with these conditions are typically required to attend more office visits for BP monitoring.

The objective of this article is to examine the feasibility and patient acceptability of implementing a self-monitoring BP program to remotely monitor pregnant women at higher risk for pregnancy-induced HTN during the third trimester. Pregnant women residing in rural medically underserved areas experience a higher degree of access to care difficulties, whereby remote self-monitoring of BP may be especially advantageous for the management or early detection of HDP.

## Materials and Methods

### Study design

We implemented a prospective repeated measures single-cohort study to examine the feasibility of a self-monitoring protocol for remote collection of BP readings and HDP at a rural health clinic.

### Study setting

We partnered with the Mountain Area Health Education Center (MAHEC), a rural health clinic that serves the entire 16 county region of western North Carolina (WNC) and is the state's largest area health education center. An estimated one-in-five residents (∼2.2 million people) in North Carolina live in a rural under-resourced county.^11^ WNC is a particularly rural portion of the state and has been characterized as a health professional shortage area. The area has historically high rates of preterm birth and low birth weight infants compared with national averages.^12,13^ As a whole, this segment of the population is underrepresented in research in general, particularly concerning the implementation of mobile health technology to reduce maternal health disparities.

### Participants and recruitment

The inclusion criteria for pregnant women to participate in our study were to be between 26 and 40 weeks of gestation, attending an MAHEC centering group, and who owned a smartphone and they were invited to participate in the study. To test the feasibility of our protocol, we did not restrict our sample to only women at risk for high BP. Centering Pregnancy is a group-based prenatal care model wherein women regularly convene to learn good prenatal health and infant health practices. Research demonstrates the efficacy of a group prenatal care model (e.g., Centering Pregnancy) in significantly dissolving health disparities and improving perinatal health outcomes, patient satisfaction, patient and provider communication, and prenatal education.^14–17^

Recruitment took place from June 2019 to March 2020. The research team worked closely with the Centering Pregnancy team to identify 8–12 centering groups (12–15 women in each group) for targeted recruitment. Women with serious arrhythmia, severe blood flow problems, or blood disorders, as well as complicating factors such as common arrhythmias, ventricular premature beats, atrial fibrillation, arteriosclerosis, poor perfusion, diabetes, preeclampsia or renal disease are at a higher risk for obtaining a BP reading error and were excluded from participation in this study.

Upon consent, women were asked to complete a short 31-item baseline survey asking about background demographic information, before pregnancy health, prenatal health and access barriers, e-health literacy,^18^ and new uses of digital devices during pregnancy. Participants were then instructed on how to use the at-home BP instrument and app to record their BP measures twice daily (morning and afternoon).

### Home BP monitor

**FIG. 1.**
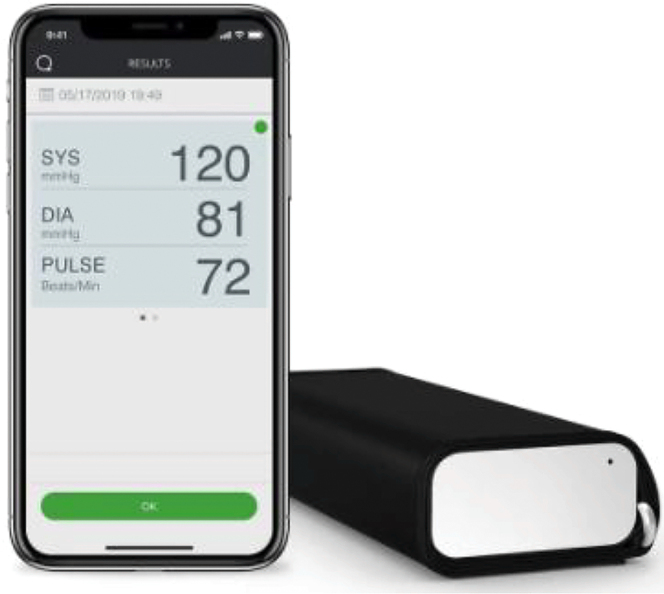
QardioArm smartphone app and BP cuff. BP, blood pressure.

The QardioArm portable BP monitor and smartphone app were used in this study to measure BP (Fig. 1). QardioArm is a U.S. Food and Drug Administration (FDA) approved and clinically validated portable BP monitor that obtains a triple measurement average for accuracy and integrates with Apple and Android devices. The instrument includes a smartphone application for BP collection and includes an option to be connected to a physician remote monitoring platform for secure communication called QardioMD. The QardioMD is a HIPAA-compliant smart platform that allowed the research team to remotely monitor BP and heart rate without requiring participants to send data separately.

The study protocol was approved by the university's institutional review board (protocol no. 14352).

### Study outcomes

The primary study outcomes examined were(1) retention and persistence of weekly BP monitoring during late-stage pregnancy, (2) differences between weekly self-monitored and clinic BP measures, and (3) the performance of self-monitored BP in early detection of pregnancy-induced HTN compared with the clinic visit. We also examined the following secondary outcomes: (1) receptivity to m-health technology for prenatal monitoring, (2) e-health literacy, and (3) prenatal care utilization and satisfaction.

American College of Obstetricians and Gynecologists (ACOG) guidelines were used to define gestational HTN and preeclampsia (ACOG 2019). Continuous systolic (SP) and diastolic (DP) BP measures were grouped into the following four categories: low (systolic ≤85), normal (systolic 86–139 and/or diastolic ≤90), raised (systolic 140–159 and/or diastolic 91–109), or alert physician (systolic ≥160 and/or diastolic ≥110). Mean arterial pressure (MAP) is a useful physiological marker to screen for preeclampsia during pregnancy.^19–22^ MAP was calculated for each clinic and home BP reading wherein both systolic and diastolic pressures were recorded using the following formula: MAP = DP +1/3(SP−DP) or MAP = DP +1/3(pulse pressure).^23^ All daily BP readings were averaged to derive one representative reading for systolic, diastolic, and MAP readings.

### Statistical analysis

Descriptive statistics were performed to analyze patient characteristics and survey responses using mean (standard deviation [SD]), median (interquartile range), and frequency (percentage) where appropriate. To assess the comparability between the clinic and home BP measures, we calculated the Bland–Altman agreement to determine whether the two methods are comparable enough that the home BP might replace the clinic measure with sufficient accuracy.^24^ We generated Bland–Altman plots to visualize the difference between the two methods (M1_clinic_−M2_home_) against the average (M1_clinic_−M2_home_)/2 with a horizontal line at M1−M2 (solid blue line), the mean of the difference (M1_clinic_−M2_home_) (solid red line), and dotted lines at ±2 SD.^25^ We relied on the 2 SD limits (dashed red lines) to demonstrate where 95% of the differences between the two methods should fall if the differences were normally distributed. This measure generates a mean discrepancy measure and is reflective of systematic bias, and the 95% limits of agreement reveal discrepancies likely to be clinically relevant in practice. We examined the test for zero bias using a paired *t*-test to indicate further whether there was bias between the two methods (*α* = 0.05). All data were analyzed using SAS version 9.4 (SAS Institute, Inc., Cary, NC).^26^

## Results

A total of 30 women consented to participate in our study (*n* = 3 missing survey data, *n* = 3 loss to follow-up, *n* = 4 remote data collection issues), and 23 participants completed both the survey and home BP monitoring portion of the study. The majority of women in our study were white, married, spoke English, and were at the beginning of their third trimester (Table 1). There was an even blend of women in the “18 to 29” and “30 to 39” age groups, and a roughly equal proportion of women with a “high school degree or less” or a “college degree.”

**Table 1.** Maternal Demographic Characteristics

	*n* (%)^a^
Mean age in years (SD)	30.1 (6.3)
Age categories	
18–29 years	13 (48)
30–39 years	14 (52)
Race	
White	21 (75)
Black	2 (7)
Other	4 (14)
Ethnicity	
Hispanic/Latinx	1 (4)
Not Hispanic/Latinx	26 (96)
Education	
Some high school	1 (4)
High school diploma/GED	11 (41)
Associates	3 (14)
Bachelors	6 (21)
Graduate/professional	6 (21)
Relationship status	
Married	20 (74)
Separated	1 (4)
Unmarried relationship	6 (22)
Primary language	
English	26 (96)
Spanish	1 (4)
Median week of pregnancy (IQR)	32 (31, 34)

^a^ *n* = 27 participants completed the baseline survey.

GED, General Education Development; IQR, interquartile range; SD, standard deviation.

### Prepregnancy health

A total of 19% of women (*n* = 5) reported not being in regular contact with a health care provider during the 12 months before becoming pregnant. Pre-existing conditions included two (7%) reported high BP or HTN and five (19%) reported depression. A total of one out of five women reported visiting a hospital or emergency room due to a pre-existing condition followed by 52% of women (*n* = 14) reported a regular check-up at a family doctor's office, 33% reported a health care visit for a chronic condition, and 26% (*n* = 7) reported a health care visit for anxiety or depression. Roughly 33% of participants (*n* = 9) reported quitting smoking.

### Prenatal health

New health conditions arising during the current pregnancy included three women (11%) self-reported type 1 or 2 diabetes, three women (11%) reported high BP or HTN, and three women (11%) reported depression. A total of six women (22%) reported a visit to the hospital or emergency room resulting from these health conditions, half of whom reported HTN. In general, women in our sample were satisfied with their prenatal care, including satisfaction with the (1) amount of time they had to wait (*n* = 21, 78%), (2) amount of time with their provider (*n* = 25, 93%), (3) prenatal education (*n* = 25, 93%), and (4) understanding and response shown by their provider (*n* = 25, 93%). The majority of respondents were able to obtain prenatal care as early during their pregnancy as they wanted (*n* = 26, 96%), and 85% of women (*n* = 23) were able to attend all of their recommended prenatal visits.

Reported barriers to care were low, but the top three barriers to receiving prenatal care were did not have enough money or insurance to pay for visits (*n* = 3, 11%), could not get to an appointment when I wanted to (*n* = 2, 7%), and could not take time off from work or school (*n* = 2, 7%).

### New uses of digital devices during pregnancy

**FIG. 2.**
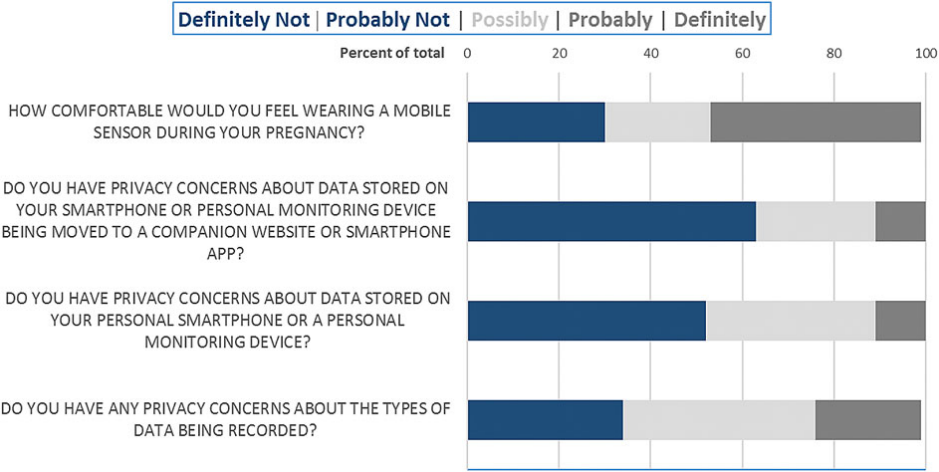
Participants self-reported level of comfort with data collected using m-health technology during pregnancy.

All women in our study reported having access to the internet and most reported using it every day (89%, *n* = 24). When asked whether or not participants would be willing to wear a GPS tracker while pregnant, the majority responded *sometimes* (*n* = 11, 41%) followed by *often* (*n* = 5, 19%) and *all the time* (*n* = 5, 19%). Participants were split when responding they would wear a GPS tracker *only certain times of the day* (*n* = 8, 30%) or *all day* (*n* = 8, 30%), whereas many were agreeable to wearing a geolocational tracker for their *entire pregnancy* (37%, *n* = 10). Women did express some privacy concerns about the types of data being recorded when using personal devices with embedded sensors or sensing capabilities (42%, *n* = 11 responded “possibly concerned”) (Fig. 2). Respondents were mostly comfortable with moving data stored on their smartphone or personal monitoring device to a companion website or smartphone app (63%, *n* = 17 responded “definitely not or probably not” to privacy concerns). However, women expressed being more comfortable with wearing a mobile sensor during pregnancy (46%, *n* = 12 responded “probably or definitely”).

**FIG. 3.**
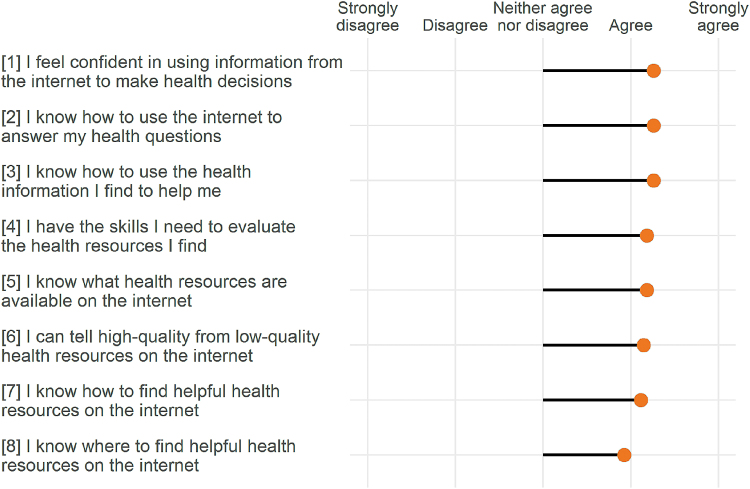
Mean e-health literacy scores (−2 strongly disagree, −1 disagree, 0 neutral, 1 agree, and 2 strongly agree).

Women reported high health-related internet use across all search categories with the most frequent searches for the following topics: (1) healthy lifestyle (96%, *n* = 26), (2) care providers (93%, *n* = 25), and (3) medical treatments (89%, *n* = 24). Similarly, participants also reported high e-health literacy (Fig. 3). Roughly 9 out of 10 women (93%, *n* = 25) reported a willingness to change their behavior during pregnancy in response to receiving personalized recommendations from a smartphone.

### Home monitoring

Women enrolled contributed an average of 31.2 days (SD 27.0) of home BP monitoring. A total of two women had previously been diagnosed with chronic HTN before pregnancy, and two women in our study experienced a hypertensive disorder of pregnancy (*n* = 1 gestational HTN, *n* = 1 preeclamptic with severe features) (Table 3). For one woman, we were able to detect a raised BP using the home remote monitoring system 1 week before it was detected at the clinic. Another participant in our sample reported replacing her smartphone a few weeks into the study but persisted using the QardioArm BP cuff to monitor her BP daily. As a result, she caught her BP raising significantly, notified her physician, and was induced early due to preeclampsia.

### Comparison between BP collection methods

**FIG. 4.**
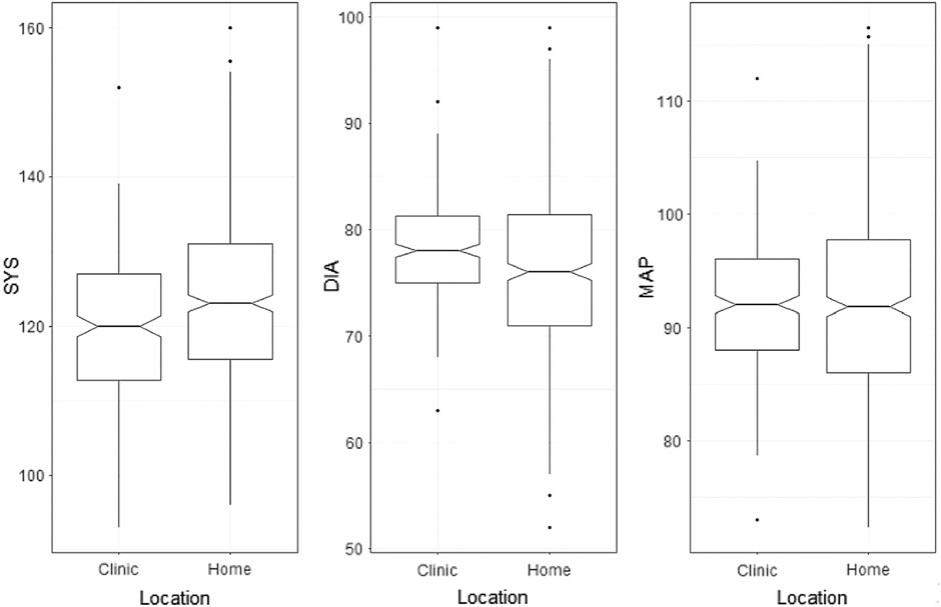
The differences between clinical and home recordings of BP for systolic, diastolic, and MAP measurements. MAP, mean arterial pressure.

Table 2 provides a descriptive overview of the clinic and home BP measures (Supplementary Fig. S2). In general, Bland–Altman plots revealed that home BP systolic measures tended to be higher on average than those collected at the clinic (Supplementary Fig. S1). This was confirmed with a statistically significant paired *t*-test for zero bias (*t*_(271)_ = −7.04, *p* < 0.0001), signifying that there was a bias between the clinic and home BP systolic measures. Home BP diastolic measures were slightly lower than clinic measures (Supplementary Fig. S2) and this bias was confirmed with a statistically significant paired *t*-test for zero bias (*t*_(271)_ = 2.47, *p* < 0.01). However, we found no difference between MAP measures derived using clinic data and those derived from home recordings of BP (Supplementary Fig. S2, paired *t*-test for zero bias (*t*_(271)_ = −1.89, *p* = 0.06). Figure 4 illustrates the differences between clinical and home recordings of BP for systolic, diastolic, and MAP measurements.

**Table 2.** Descriptive Statistics for Blood Pressure Measures Obtained at the Clinic and Obtained Remotely from Home Blood Pressure Collection

	Clinic BP measures	Home BP measures
Systolic		
Mean (SD)	120.3 (10.5)	124.9 (10.6)
Median (25th, 75th)	120.0 (112, 127)	124 (118, 131)
IQR	14.5	13.2
Diastolic		
Mean (SD)	78.4 (6.0)	77.4 (7.1)
Median (25th, 75th)	78.0 (75, 82)	77.6 (72, 82)
IQR	6.5	9.2
MAP		
Mean (SD)	92.4 (7.1)	93.2 (7.4)
Median (25th, 75th)	92.0 (88.0, 96.0)	92.7 (88.4, 97.7)
IQR	8.0	9.3
BP		
Low	161 (44.2)	0
Normal	202 (55.9)	442 (98.2)
Raised	1 (0.3)	8 (1.7)
Alert	0	0

BP, blood pressure; MAP, mean arterial pressure.

**Table 3.** Participant Outcomes

	N = 30
Total participants who contributed to remote home BP monitoring	23 (76.6)
*n* = 3 loss to follow-up	
*n* = 4 equipment failure	
Total participants with provider-diagnosed chronic hypertension	2 (7.4)
Total participants with provider-diagnosed hypertensive disorders of pregnancy	2 (7.4)
Total participants with at least one “raised” home BP measures	8 (26.7)
Total participants presented with more than one “raised” home BP measures^a^	2 (7.4)
Total participants with “raised” clinic BP measures	1 (3.7)
Days of home BP monitoring	
Mean (SD)	16.2 (12.8)
Median (25th, 75th)	12.5 (6, 25)
IQR	19
Missed clinic appointment	
Mean (SD)	5.1 (2.9)
Median (25th, 75th)	5 (3, 7)
IQR	4
Returned home BP monitor	13 (48.1)

^a^ *n* = 1 replaced home BP monitor due to higher-than-normal BP readings.

## Discussion

Results from this study demonstrate the feasibility of implementing home BP monitoring for pregnant women in a rural care setting. Our sample of pregnant women was a diverse blend of age groups and educational backgrounds. A high proportion of women reported a pre-existing chronic health condition and 22% visited an emergency department for a new health condition diagnosed during their current pregnancy. Although these women reported high satisfaction with prenatal care, on average, this group missed 5 out of 13 clinic visits (54%) during the third trimester. Conversely, we observed high participation in home BP monitoring in which women logged an average of 16 daily (range 5 days to 4 weeks) BP readings during the final weeks of their pregnancy.

All women reported having access to the internet. Nearly half of the participants were willing to wear a mobile sensor during pregnancy, and one in five were willing to wear a mobile sensor for their entire pregnancy. Lastly, findings showed that home systolic and diastolic BP readings varied from clinic readings. Still, the difference did not exceed >2 SDs above the mean for clinic visits and suggests that prenatal home BP monitoring may be used in early detection of anomalous BP readings. Although preliminary, we confirmed that remote monitoring can detect elevated BP earlier than in routine clinic visits. To our knowledge, this is one of the first studies to examine home BP monitoring during pregnancy in rural Appalachia.

In our patient population and among rural women, more generally, low health and technology literacy have been cited as important barriers to clinical implementation of technology-enabled care models.^27,28^ However, our survey findings revealed high health-related internet use and e-health literacy. Furthermore, low health literacy has been associated with increased ED use, fewer scheduled preventative health visits, lower levels of health information technology use, and an elevated risk for costly disparities in health outcomes, and in severe cases, death.^29,30^ Along with the emergence of technology-enabled health care has arisen the need to bolster e-health literacy defined as the ability to look for, identify, understand, and act on health information from online sources.^31^ Health literacy is a key determinant of health.^32^ As the medical community advances toward patient-centered care that relies heavily on self-monitoring technology, the role of health literacy or e-health literacy will be pivotal in preventing digital disparities.^33^ Recent studies have shown that among individuals with low health literacy, mobile apps are an acceptable platform for individuals to obtain, interact with, and use in their health care.

Earlier research revealed wide individual-level variation in BP levels recorded at home compared with those using the gold standard of the mercury sphygmomanometer.^34^ A recent study examined the application of remote BP monitoring for postpartum HTN management, and similar to our study demonstrated high retention rates, early identification of severe BP, and a reduction in hospital readmissions for HTN after the delivery discharge.^35^ Some research has cautioned about the concern for heightened anxiety, especially among women with high BP, due to a false raised reading. Although only two women in our study were diagnosed with a hypertensive disorder of pregnancy, home monitoring identified eight women with a raised BP reading. But upon being prompted to record another measure, only two women with more than one elevated BP reading were identified, lending to the accuracy of the FDA-approved QardioArm BP cuff. Although few studies have focused on home monitoring of BP during pregnancy, none have thus far demonstrated adverse outcomes.^35–38^

### Strengths and limitations

Clinic-based assessment of BP is not without limitations, including white coat syndrome, observer error, improper sizing of the cuff, rounding off measures, and observer bias with a tendency toward “normalizing BP.”^39^ Although results showed variability between BP readings obtained at the clinic and by way of self-monitoring, we assert that home BP measures might be more accurate and representative of the “true” daily BP levels for women in our study. We also assert that home BP monitoring provides a more cost-effective and noninvasive means for women to monitor their BP during late-stage pregnancy, particularly those in rural areas. An additional strength of our study is the prospective measurement and remote collection of multiple BP readings throughout the third trimester.

There were a few limitations in this study, including the small sample size of women in advanced pregnancy (the mean gestational week was 33) and the lack of comparative results from home and clinic measures collected at or around the same time. A number of women in our study were willing to undergo health monitoring for the duration of their entire pregnancy, and future studies should examine both pre- and postpartum changes in BP. Although women reported that the smartphone app was easy to use, a few reported the BP cuff was too tight and uncomfortable (*n* = 3). Additional iterations of the protocol should include cuff sizing. Another limitation is that we only collected data on whether or not a woman had a pre-existing or newly diagnosed chronic condition (e.g., type 1/type 2 diabetes, high BP or HTN, and depression) that may be associated with medication use that could affect BP. Although only two participants (8.7%) recorded high BP readings, this number is reflective of national estimates showing that high BP or related conditions (e.g., HDP, eclampsia) occur in 6–8% of pregnancies.

### Future research

**FIG. 5.**
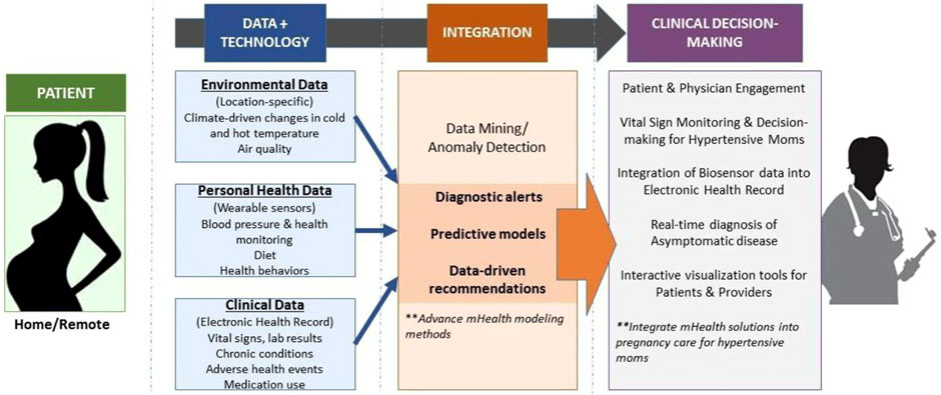
Conceptual flowchart of data streams for m-health applications for pregnant women.

More research is needed to understand how well data collected from at-home monitors predict adverse pregnancy-related blood pressure issues, as well as how receptive patients and providers are to using these technologies to monitor pregnancy. Ultimately we propose the use of multiple data streams during pregnancy that can be integrated into predictive models to achieve data-driven clinical decision making for pregnant patients with hypertensive disorders (Fig. 5). Before the widespread use of remote monitoring is integrated into routine clinical practice, more rigorous clinical evaluation of m-health innovations is required.^40^ Validation studies on the predictive value of self-monitoring during pregnancy as a screening tool for early detection of HTN disorders of pregnancy are needed that shed insight into how to account for elevated BP during the last few weeks of pregnancy, as well as how to integrate the diagnostic capabilities of BP self-monitoring protocols as a routine part of prenatal care coordination. Prospective studies incorporating a randomized-control trial or hybrid effectiveness-implementation design^41,42^ that include large samples of pre- and postnatal women are needed, particularly among women at risk for HTN.

### COVID-19 pandemic and prenatal care

Pregnant women will continue to need access to safe accessible prenatal care for the duration of this pandemic. The current COVID-19 pandemic has further highlighted the importance of integrating effective remote health monitoring technology into routine prenatal care. Pregnant women may face disproportionately higher risks of morbidity and mortality from viral respiratory illnesses such as COVID-19 due to changes in cardiorespiratory and immune systems during pregnancy.^43,44^ Widespread closures or reduced service hours of health care providers have limited access to prenatal care, and fear of exposure to the virus has made patients more reluctant to seek medical care for routine or emergency visits. For pregnant women specifically, the pandemic may amplify existing barriers to receiving prenatal care including lack of child care, loss of income, or lack of social support networks. Routine prenatal visits are essential for monitoring maternal and fetal health—and this is especially true now when novel COVID-19–related complications may arise during pregnancies. Transitioning to digital health technology allows health care providers to continue offering physically distanced prenatal care during a pandemic situation when in-person visits are unfeasible or undesirable (e.g., Aleha et al.^19^).

## Conclusion

Wearable technologies can provide low-cost improvements to patient–provider interactions and remote monitoring of pregnancy health management, especially for rural underserved populations. The integration of cost-effective remotely accessed devices in obstetric care provides a scalable platform for engaging with and caring for pregnant women in rural Appalachia.

## Supplementary Material

Supplemental data

Supplemental data

## Abbreviations Used

ACOG: American College of Obstetricians and Gynecologists
BP: blood pressure
DP: diastolic pressure
FDA: U.S. Food and Drug Administration
GED: Graduate Equivalence Degree
HDP: hypertensive disorders of pregnancy
HTN: hypertension
IQR: interquartile range
MAHEC: Mountain Area Health Education Center
MAP: mean arterial pressure
SD: standard deviation
SP: systolic pressure
WNC: western North Carolina
