# Supplementary material for: Examining the Feasibility of Smart Blood Pressure Home Monitoring: Advancing Remote Prenatal Care in Rural Appalachia
Source: Telemed Rep. 2021 Mar 24;2(1):125–34. doi: 10.1089/tmr.2020.0021 (PMC9049804; doi:10.1089/tmr.2020.0021)
Supplement: Supplemental data [file Supp_Fig2.docx]

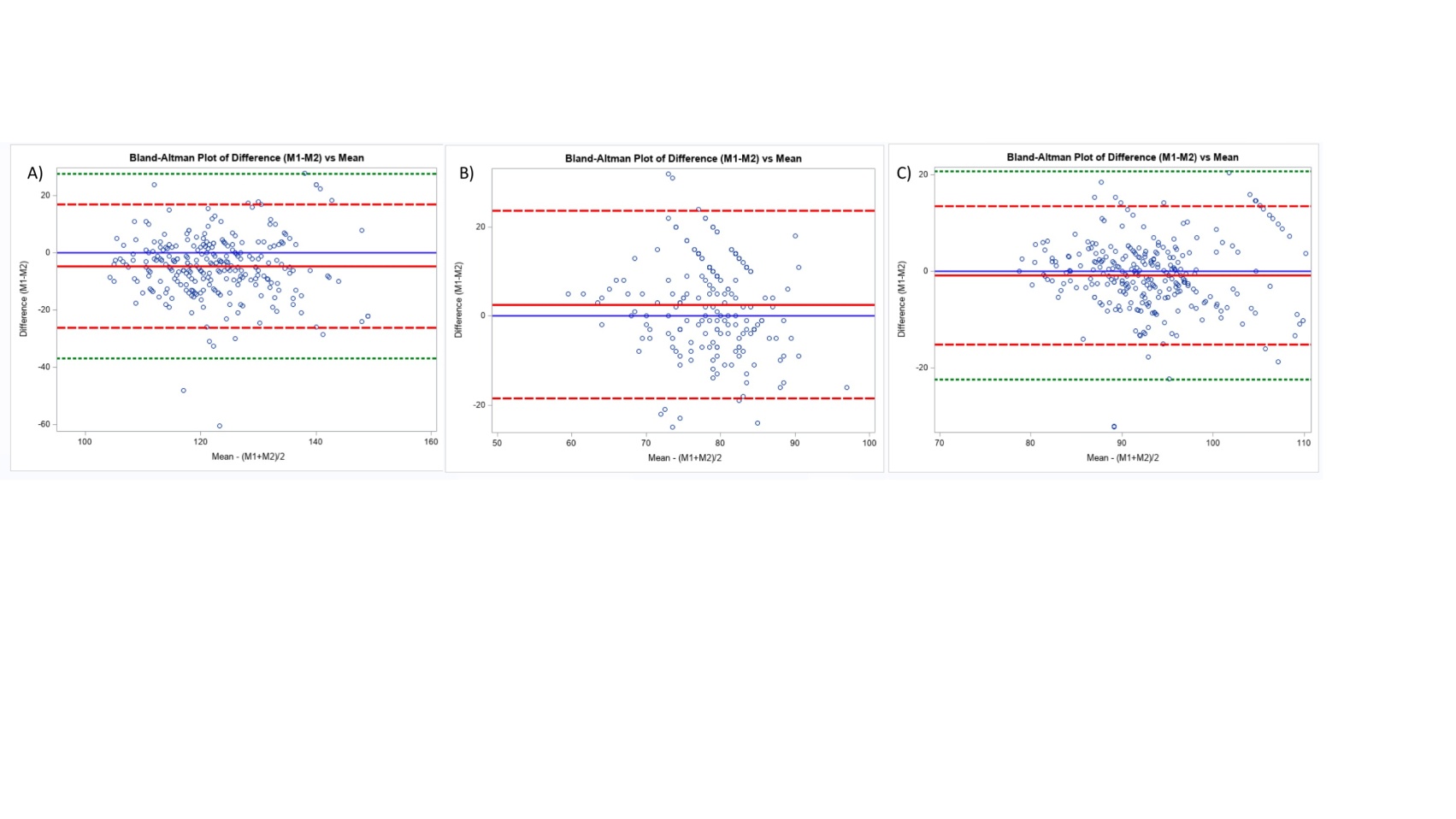


Supplemental Figure 2. Bland-Altman plot of the difference (M1_clinic_-M2_home_) vs. the mean (M1_clinic_+M2_home_)/2 of the clinic and home BP methods of measurement for a) *systolic* blood pressure, b) diastolic blood pressure, and c) mean arterial pressure (MAP).
