# Supplementary material for: Examining the Feasibility of Smart Blood Pressure Home Monitoring: Advancing Remote Prenatal Care in Rural Appalachia
Source: Telemed Rep. 2021 Mar 24;2(1):125–34. doi: 10.1089/tmr.2020.0021 (PMC9049804; doi:10.1089/tmr.2020.0021)
Supplement: Supplemental data [file Supp_Fig1.docx]

**Supplemental Materials**

**
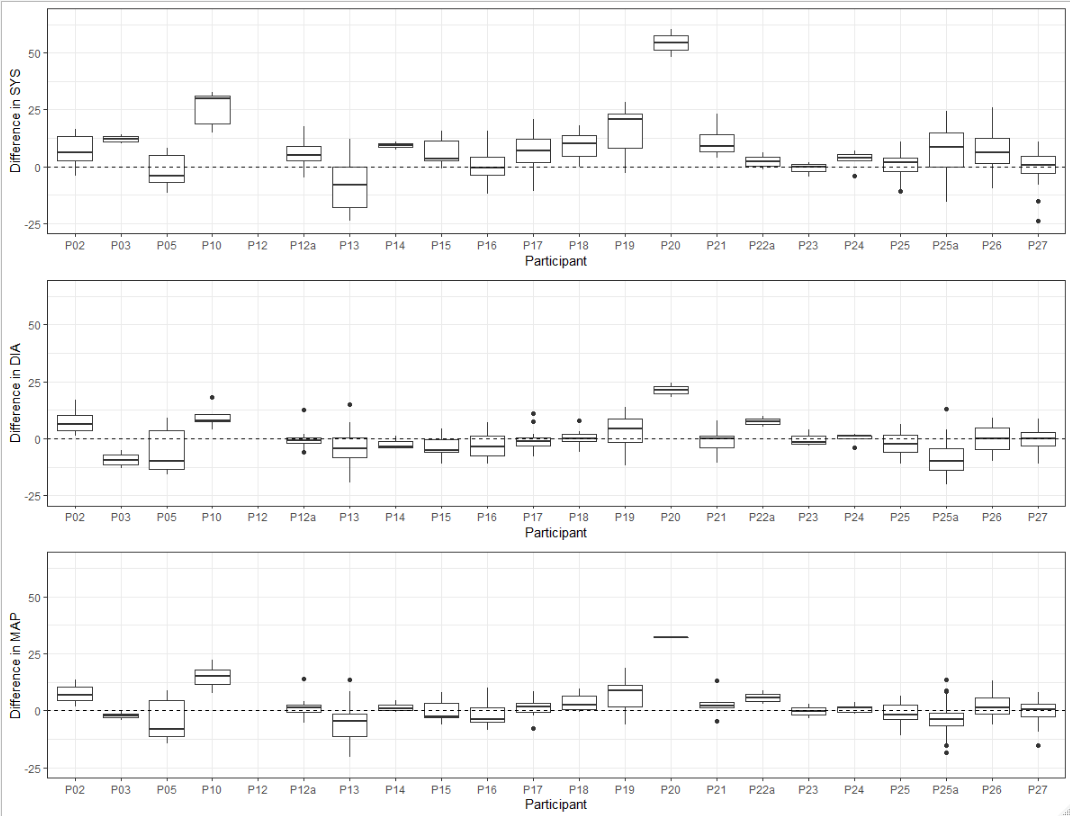
**

**Supplemental Figure 1:** The differences between clinical and home recordings of BP for systolic, diastolic, and MAP measurements for each participant.
